# Hypoglossal nerve trunk stimulation: electromyography findings during drug-induced sleep endoscopy: a case report

**DOI:** 10.1186/s13256-023-03877-2

**Published:** 2023-05-06

**Authors:** E. R. Thuler, F. A. W. Rabelo, Vanier Santos Junior, F. Kayamori, E. M. G. Bianchini

**Affiliations:** 1 University of Pennsylvania Perelman School of Medicine, 3400 Spruce Street, Ravdin 5, Philadelphia, PA USA; 2 Hospital Samaritano, São Paulo, Brazil; 3 University of São Paulo Medical School, São Paulo, Brazil; 4 Pontifical Catholic University of São Paulo, São Paulo, Brazil

**Keywords:** Obstructive sleep apnea, Upper airway stimulation, Hypoglossal nerve stimulation, Electromyography

## Abstract

**Background:**

Literature has demonstrated hypoglossal nerve stimulation to be a safe and effective treatment for patients with obstructive sleep apnea nonadherent to positive airway pressure therapy. However, the recommended criteria for patient selection are still unable to identify all the unresponsive patients, highlighting the need for improved understanding about hypoglossal nerve stimulation for obstructive sleep apnea.

**Case presentation:**

A 48-year-old Caucasian male patient with obstructive sleep apnea had been successfully treated with electrical stimulation of the hypoglossal nerve trunk, documented by level 1 polysomnography data. However, due to snoring complaints, he underwent postoperation drug-induced sleep endoscopy for evaluation of electrode activation during upper airway collapse, aiming to improve electrostimulation parameters. Concurrent surface electromyography of the suprahyoid muscles and masseter was obtained. Activation of electrodes 2, 3, and 6 promoted upper airway opening most strongly at the velopharynx and tongue base during drug-induced sleep endoscopy. The same channels also significantly increased the electrical activity on suprahyoid muscles bilaterally, but predominantly on the stimulated side (right). The masseters also presented a considerable asymmetry in electrical potential on the right side (> 55%).

**Conclusion:**

Beyond the genioglossus muscle, our findings demonstrate recruitment of other muscles during hypoglossal nerve stimulation, which may be attributed to the electrical stimulation of the nerve trunk. This data provides new insights on how stimulation of the hypoglossal nerve trunk may contribute to obstructive sleep apnea treatment.

## Background

Hypoglossal nerve stimulation (HGNS) can increase genioglossus muscle activity and effectively prevent pharyngeal collapse during sleep in patients with obstructive sleep apnea (OSA) [1]. In the past three decades, several studies have demonstrated HGNS to be a safe and effective treatment for patients with OSA not adherent to positive airway pressure (PAP) therapy [2]. There are currently several types of devices differing regarding implantation site and stimulation mode [3]. Ultimately, OSA severity defined as Apnea–Hypopnea Index (AHI) > 65, body mass index (BMI) > 35, and complete circumferential pattern of collapse during drug-induced sleep endoscopy (DISE) were considered exclusion criteria for HGNS implantation [4, 5]. However, the success rate among patients meeting criteria for HGNS therapy (60–70%) suggests the need for further understanding about this therapy [6].

Surface electromyography (EMG_s_) is a noninvasive method to detect and record electric potentials from voluntary muscles. This enables analysis of the myoelectric signal generated by physiological changes in the muscular fibers’ membranes. It has been established as an important method for investigating the neuromuscular system, and as a tool for clinical evaluation and applied research. The measured potentials represent the relative level of recruitment in a motor unit underlying the electrodes. Using EMG_s_, insights have been gained into the understanding of intentional and reactive motor behaviors, as well as the involvement of secondary responses to targeted neurostimulation [7].

The objective of this case report is to use EMG_s_ to analyze the effect of selective stimulation of the hypoglossal nerve trunk on suprahyoid and masseter muscle responses, and airway collapse during DISE. Our hypothesis is that selective neurostimulation of hypoglossal nerve trunk produces functional activation, not only of the genioglossus muscle, but also of the suprahyoid muscles on the stimulated side, providing additional contribution to therapeutic success.

## Case presentation

A 48-year-old Caucasian male, with chief complaints of daytime sleepiness, concentration difficulties, and choking during sleep came to a PAP-alternative clinic to evaluate candidacy for HGNS. The symptoms started in 2010 and have progressively worsened. The patient’s palatine tonsils were removed during childhood, and a septoplasty was performed in 2012 with no improvement in symptoms. He had no personal or family history of diseases or psychological disorders. In 2013, he was diagnosed by a level 1 polysomnography (PSG) with moderate OSA (Table 1) and tried PAP therapy unsuccessfully. Mandibular advancement device (MAD) was tried and provoked temporomandibular joint pain.

**Table 1:** Polysomnography data: preoperative data, 4 months postoperative data, 12 months postoperative data

PSG	Preoperative	4 months Postoperative (10/29/2015)	17 months postoperative (22/11/2016)
Sleep efficiency (%)	75.4 (406 minutes)	83.9 (360 minutes)	86.9 (443 minutes)
Arousal Index	35.8 (200)	16.5 (99)	7
N1 (%)	1.9	1.7	16.4
N2 (%)	32.6	77.6	72.8
N3 (%)	49.2	20.7	6.9
REM (%)	16.2	0	4
AIH (events/hour)	24.1	7	3.9
AI (events/hour)	9.7	0	1.5
AIH REM (events/hour)	52.8	7	3.4
AIH NREM (events/hour)	18.6	7	3.9
IDR	24.1	7	3.9
O_2_ (%) Nadir	96	94	95
Minimum SpO_2_ (%)	92	89	89
T < 90% (seconds)	0	0.1	0.1

N1,N2, N3 and REM are sleep stages

*AIH = AHI* Apnea/hipopnea index,* NREM* non REM sleep,* IDR = RDI* Respiratoty dessaturation index,* T < 90* time under 90%,* O*_*2*_ saturation

**Fig. 1:**
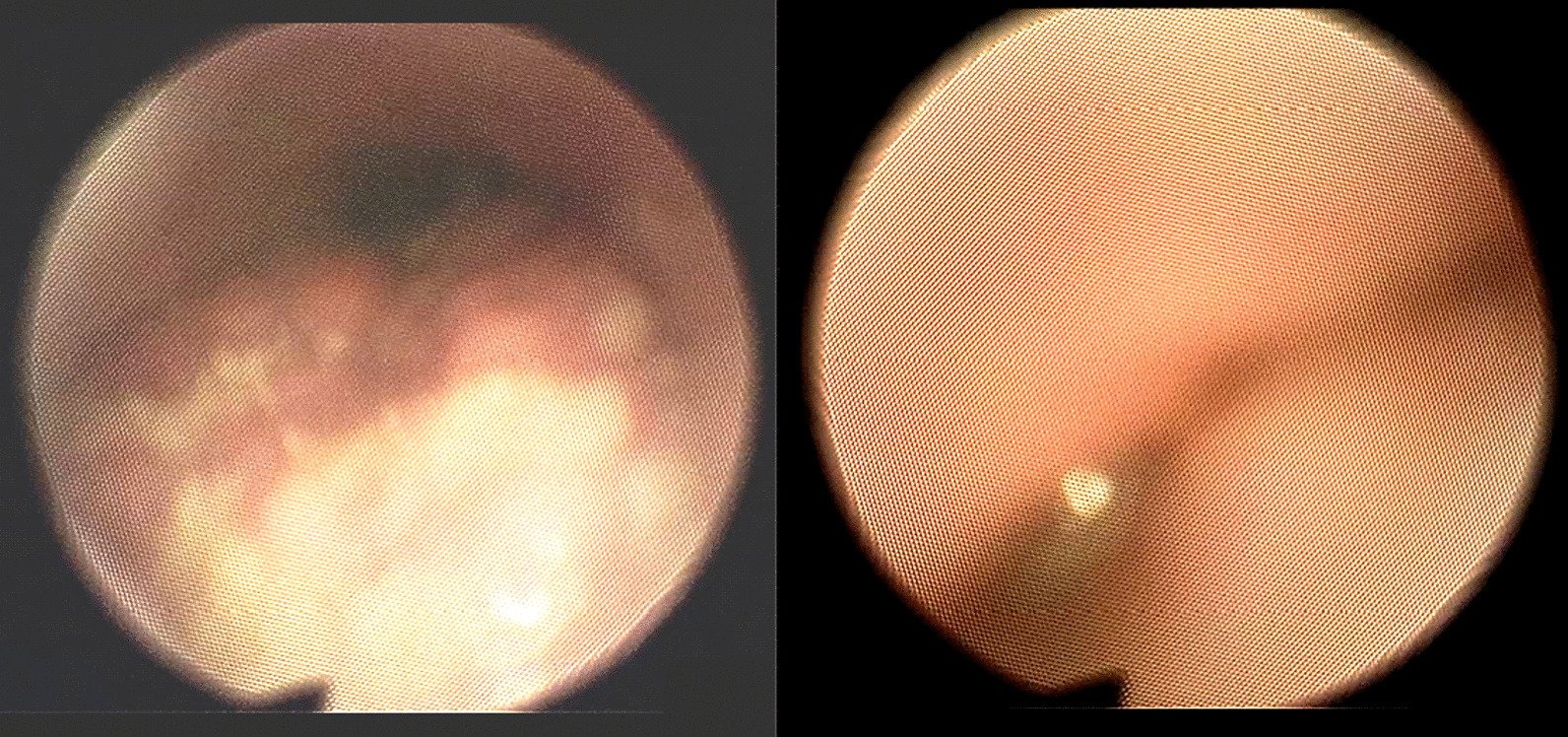
DISE demonstrating complete (VOTE grade 2) tongue base (left) and palatal (right) collapse

This patient had a BMI of 19.8, weighed 156.6 pounds (71 kg), and was 5′7″ feet tall (1.71 m). Physical exam revealed a Friedman Tongue Position grade 4 [8], and a narrow hard palate (posterior crossbite). No other craniofacial abnormalities were detected. Drug-induced sleep endoscopy (DISE) was performed in 2014 to confirm candidacy for HGNS, detecting complete [velum, oropharynx, tongue and epiglottis (VOTE) grade 2] velopharyngeal anteroposterior collapse and tongue base collapse (Fig. 1).

Implantation of HGNS was proposed. The procedure was performed in June 2015, and as the procedure was not covered by insurance, the patient paid out of pocket. The ImThera (aura 2000) device was selected for implantation once it was approved under special conditions in Brazil. The device was turned on 1 month after the procedure, after determination of motor and sensory thresholds, followed immediately by a titration PSG. Based on the effect on airflow and sleep parameters, electrodes 2, 3, and 5 were selected for therapy. Clinical improvement was reported by the patient and confirmed by PSGs performed at 4 and 17 months after the procedure (Table 1).

**Fig. 2:**
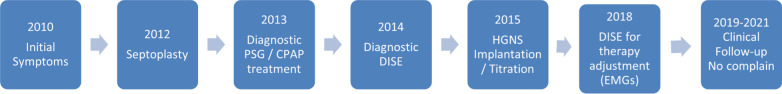
Case report Timeline

In 2018, due to snoring complaints, a second DISE was performed to evaluate and improve electrostimulation parameters. The exam was conducted under propofol sedation using target-control-infusion (TCI) and bispectral index monitoring (BIS) [9]. Informed consent and institutional research ethics board (IRB) approval were obtained from Pontifical Catholic University of São Paulo (PUC/SP) (REB protocol 1.964.298). Superficial electromyography (EMG_s_) of the suprahyoid muscles and masseters was recorded simultaneously during DISE to evaluate the effectiveness of each contact electrode on muscular response using the previously published Miotool Face USB (Miotec) protocol [10–12]. Asymmetric muscular response was defined as greater than 20% discrepancy between sides [10, 11] (Fig. 2).

Initially, baseline electromyographic activity was obtained while awake, and continued during DISE, with HGNS turned off and on. First, the recording was obtained while the patient was seated, and low potential activity was observed in both the suprahyoid muscles and masseters, symmetrically. Second, maximum voluntary suprahyoid muscle contraction was evaluated by instructing the patient to hold the mouth open, generating electrical potentials that were 29.50% greater on the right side. The maximum masseter response was then tested by having the patient grind their teeth against a cotton roller, and the electric potential presented a response 19.69% greater on the right side. In the supine position, the activity of the suprahyoid muscles increased by approximately 100% bilaterally, while the masseters activity increased around 70% only on the right, representing an asymmetric response (Table 2).

**Table 2:** Surface electromyography results for the muscles, namely the masseters and suprahyoid group, prior to electrostimulation, with the patient awake and seated, awake and laying down, and sedated

	Right masseter (RM)	Left masseter (LM)	Difference LM × RM %	Right suprahyoid (RS)	Left suprahyoid (LS)	Difference LS × RS %
Rest closed lips: awake and seated (µv)	4.2	5.0	15.6	7.2	6.3	11.3
Normalized (%)	1.3	1.9	32.2	4.6	5.7	20.5
Isometric contraction: awake and seated (µv)	200.0	186.6	6.7	17.4	13.1	24.2*
Normalized (%)	61.8	71.8	13.9	11.1	11.9	7.0
Maximum isometric contraction: awake and seated (µv)	293.0	269.8	7.9	10.9	11.9	8.6
Normalized (%)	90.6	103.8	12.8	6.9	10.8	35.5
Mandibular maximum opening: awake and seated (µv)	8.8	7.1	20.0*	90.6	60.5	33.3*
Normalized (%)	2.8	2.7	0.3	57.9	54.7	5.37
Rest: awake and lying down (µv)	7.2	4.9	32.3*	14.4	13.1	9.1
Normalized (%)	2.2	1.9	15.7	9.2	11.9	22.5
Rest awake: seated/lying down (%)	71.4 **	(2)	73**	100**	107**	7
Rest: Sedated/lying down (µv)	4.3	5.0	14.3	5.0	5.1	2.5
Normalized (%)	1.7	1.9	14.3	3.2	4.7	31.3
Rest sedated/awake (%)	74.82	103.5	27.7*	34.7	39.2	11.4
Calibration MVC (µv)	323.5	259.8	19.7	156.6	110.4	29.5*

Subtitles: normalized data (%), *µv* microvolts, *MVC* maximum voluntary contraction

*Electric potential difference greater than 20%, **increased % related to seated subject, (−) negative values

After starting propofol infusion, the activity of both masseters was equivalent to rest position, while the activity of the suprahyoid muscles decreased bilaterally. The activation of electrodes 2, 3, and 6 demonstrated better efficacy in upper airway stimulation during this DISE. Interestingly, electrode five was originally selected for therapy on the basis of the postoperative PSG. After stimulation, suprahyoid muscles significantly increased activity bilaterally, predominating on the side of implantation (right). The masseters presented a considerable asymmetry in the electric potential, greater in the right side (> 55%), while the activity of left masseter was relatively unchanged from rest (Table 3).

**Table 3:** Results from surface electromyography for the masseters and supra-hyoid group for electrostimulation of each channel during drug-induced sleep endoscopy

	Right masseter (RM)	Left masseter (LM)	difference LM × RM (%)	Right suprahyoid (RS)	Left suprahyoid (LS)	difference LS × RS (%)
Turned off (µv)	4.5	4.0	11.1	6.0	5.0	16.7
Normalized (%)	1.4	1.5	6.7	4.0	4.6	13.4
Activation channel 1 (µv)	16	6.2	61.3*	92.4	70.8	23.4
Normalized (%)	4.9	2.4	51.0	59	64.1	8.0
Activation channel 2 (µv)	15.5	6.3	59.4*	96.6	77.9	19.4
Normalized (%)	4.8	2.4	50.0	62.7	70.5	11.1
Activation channel 3 (µv)	15.6	6.4	59.0*	100.1	81.5	18.6
Normalized (%)	4.8	2.5	47.9	63.9	73.8	13.4
Activation channel 4 (µv)	15.7	6.3	59.9*	95.5	77.7	18.6
Normalized (%)	4.9	2.4	51.0	61	70.4	13.4
Activation channel 5 (µv)	15.4	6.4	58.4*	96.1	78.8	18.0
Normalized (%)	4.8	2.5	47.9	61.4	71.3	13.9
Activation channel 6 (µv)	15.4	6.5	57.8*	103.6	84.8	18.2
Normalized (%)	4.8	2.5	47.9	66.1	76.8	13.9

*Electric potential difference greater than 20%

## Discussion

To our knowledge, this is the first report applying EMG_s_ to monitor suprahyoid and masseter activity during HGNS, which brings new data to the field. Our results show an asymmetric increase in both suprahyoid and masseter muscular activity, lateralizing to the site of implantation. We hypothesized this to be due to electrode insertion around the hypoglossal nerve trunk, allowing a selective stimulation of branches that broadly activate muscles beyond the genioglossus to increase pharyngeal patency [13, 14].

Current literature debates the influence of electrode insertion on treatment outcomes, since results of both devices seems to be equivalent [3]. In this case, EMG_s_ analysis demonstrates a significant increase in electrical activity of both suprahyoid muscles and masseters, predominantly on the stimulated side (right), simultaneously to the stimulations of the hypoglossal nerve. Although this asymmetry was less than 20%, this result supports the hypothesis that neurostimulation of the hypoglossal trunk may produce functional activation, not only of the genioglossus muscle, but also of the suprahyoid muscle on the stimulated side [15]. We must highlight that it can also be explained by a crosstalk when the electrical activity is captured, due to the close relationship between the suprahyoid muscles and surrounding musculature. [7, 11]

This asymmetry in EMG_s_ recording was also present while the patient was awake and seated, primarily at maximal mouth opening. Because there is no occlusal interference, we expected a symmetric increase in activity of the suprahyoid muscles [16]. Considering that the patient was using the HGNS for more than one year, this result also conveys new data about this therapy, suggesting an increased recruitment of fibers on the side where neurostimulation has been performed.

Activation of the masseter muscles during stimulation of the hypoglossal nerve trunk was an unexpected result. A considerable increase in right masseter contraction (> 50%) was observed during DISE after HGNS therapy was turned on, whereas the left masseter activity remained relatively unchanged. Crosstalk is not a plausible explanation considering the distance between these muscles. We hypothesize that this coactivation of the right masseter occurred in response to a broader muscle recruitment triggered by the stimulation of the hypoglossal nerve trunk.

This case report also presents EMG_s_ as a noninvasive tool applied in the selection of electrodes optimal for therapy, as the ImThera device has six different options. In fact, the best EMG_s_ response was associated with the most upper airway (UA) opening during DISE (channels 2, 3, 6), suggesting its potential therapeutic benefit.

We recognize that EMG_s_ has limited external validity, as electric potentials measured are relative to the patient. The EMGs may also be limited by interference from adjacent musculature and the possibility of capturing potentials spanning multiple muscle groups.

## Conclusion

These data provide insight into the muscular responses that can contribute to UA patency due to HGNS. The recruitment of muscles beyond the genioglossus may represent a therapeutic advantage of the stimulation of the hypoglossal nerve trunk.

## Data Availability

Data supporting our findings are stored and available for consultation at https://1drv.ms/u/s!Ag38HazveasDvHLcol9qJ2sxm1Zt?e=zIOvIz.

## Abbreviations

HGNS: Hypoglossal nerve stimulation
HNT: Hypoglossal nerve trunk
OSA: Obstructive sleep apnea
PAP: Positive airway pressure
DISE: Drug induced sleep endoscopy
EMG^s^: Superficial electromyography
UA: Upper airway
BMI: Body mass index
AHI: Apnea–Hypopnea Index
BIS: Bispectral Index
TCI: Target control infusion
VOTE: Velum, oropharynx, tongue and epiglottis
PSG: Polysomnography
MAD: Mandibular advancement device
